# Age-related hearing loss: prevention of threshold declines, cell loss and apoptosis in spiral ganglion neurons

**DOI:** 10.18632/aging.101045

**Published:** 2016-09-23

**Authors:** Robert D. Frisina, Bo Ding, Xiaoxia Zhu, Joseph P. Walton

**Affiliations:** ^1^ Department Communication Sciences and Disorders, Global Center for Hearing and Speech Research, University of South Florida, Tampa FL, 33612, USA; ^2^ Department Chemical and Biomedical Engineering, Global Center for Hearing and Speech Research, University of South Florida, Tampa FL, 33612, USA

**Keywords:** aging, neuroscience, hearing loss, drug discovery, auditory system, neurodegeneration

## Abstract

Age-related hearing loss (ARHL) -presbycusis - is the most prevalent neurodegenerative disease and number one communication disorder of our aged population; and affects hundreds of millions of people worldwide. Its prevalence is close to that of cardiovascular disease and arthritis, and can be a precursor to dementia. The auditory perceptual dysfunction is well understood, but knowledge of the biological bases of ARHL is still somewhat lacking. Surprisingly, there are no FDA-approved drugs for treatment. Based on our previous studies of human subjects, where we discovered relations between serum aldosterone levels and the severity of ARHL, we treated middle age mice with aldosterone, which normally declines with age in all mammals. We found that hearing thresholds and suprathreshold responses significantly improved in the aldosterone-treated mice compared to the non-treatment group. In terms of cellular and molecular mechanisms underlying this therapeutic effect, additional experiments revealed that spiral ganglion cell survival was significantly improved, mineralocorticoid receptors were upregulated via post-translational protein modifications, and age-related intrinsic and extrinsic apoptotic pathways were blocked by the aldosterone therapy. Taken together, these novel findings pave the way for translational drug development towards the first medication to prevent the progression of ARHL.

## INTRODUCTION

Age-related hearing loss (ARHL – presbycusis) is the number one communication disorder and neurodegenerative disease of our aged population; and ranks with arthritis and cardiovascular diseases as one of the top three chronic medical conditions. We have learned much about the functional declines in auditory function with age. A loss of hearing sensitivity, beginning with the high pitches or frequencies, and the inability to understand speech, particularly in the presence of background noise, top the list of perceptual difficulties characteristic of old age [1]. Similar functional deficits in auditory perception occur in most aging mammals, including primates and rodents [2-6]. However, despite these advances in understanding the perceptual and physiological bases of ARHL, there are still no FDA-approved drugs on the market to treat the permanent hearing loss affecting over 40 million Americans, including presbycusis, and this dis-appointing situation is exacerbated globally. We are just reaching the point where our knowledge of the structural and molecular changes that occur in the inner ear (cochlea) with age, allow us to start formulating biomedical interventions to prevent or slow down the progression of ARHL. For example, it has recently been shown that knockout mice, deficient in an antioxidant protein, show accelerated age-linked loss of inner hair cells, which can be treated with an antioxidant, N-acetyl-cysteine (NAC) [7].

There are three dominant morphological changes that occur in the aging cochlea [8, 9]. i) *Sensory Presbycusis*: the loss of sensory hair cells, first outer hair cells, followed later by inner hair cells; ii)*Neural Presbycusis*: declines in the number of spiral ganglion cells; where the number of ganglion cells typically decreases up to 25% along the entire cochlear duct across the lifespan (e.g., [10, 11] and iii) *Metabolic Presbycusis*: atrophy of the stria vascularis of the cochlear lateral wall of scala media. The latter is linked to dysfunction of the Na-K-ATPase and NKCC1 ion channel pumps within the stria vascularis. An animal model, mice treated with ouabain, an inhibitor of Na-K-ATPase, involves both metabolic and neural presbycusis. When ouabain is applied to the cochlea it leads to Na-K-ATPase-associated apoptosis and degeneration of auditory nerve fibers or spiral ganglion cells of the cochlea [12]. The preferential loss of the low-spontaneous group of spiral ganglion neurons and declines of the endocochlear potential (EP) are related characteristics of presbycusis [13, 14]. More specifically, it has been demonstrated that the maintenance of the EP with its high potassium ion concentration requires a full complement of NKCC1 and Na-K-ATP channels in the basolateral membrane of the cochlea's specialized endolymph organ: the stria vascularis (SV) and ALD directly regulates protein expression and activity of NKCC1 channels [84]. But according to other animal model studies [13, 15, 16], the EP declines in the cochlea happen in later stages of life. Aldosterone (ALD), a steroid hormone from the mineralocorticoid family, helps determine the levels of potassium (K+) and sodium (Na+) ions, by regulating NKCC1 and Na-K-ATPase expression. Therefore, ALD is a drug candidate to treat ARHL, since it could delay or reverse stria metabolic pathogenesis.

Specifically, a noteworthy correlation between ALD serum levels and ARHL has been observed [17-20]. In addition, ALD levels decrease with age in many species including humans and mice [21-27]. As an example, we previously discovered that higher serum aldosterone (ALD) levels correlate with better hearing in aged human subjects [17]. Also, ALD has been shown to influence hearing, with oral administration reversing autoimmune hearing loss in mice, while spironolactone (an aldosterone antagonist) blocked this effect [18-20]. In light of these previous findings, in the present investigation we test the hypothesis that ALD may be beneficial for the aging auditory system through mechanisms involving improved hearing, cochlear cell preservation and mineralocorticoid receptor (MCR) upregulation. Part of the rationale is that normal activation of MCRs in neural systems leads to maintenance of neuronal function and survival (e.g., [28]). ALD-MCR-mediated effects are often independent of agonist ligands, such as in transport epithelial cells of the renal tubule, but in neuronal systems, such as hippocampal neurons, it is ligand-dependent [29, 28, 30, 31]. In sum, spiral ganglion neurons and SV cells could be regulated by ALD to modulate cell survival. The present article reports a series of experiments that investigate how ALD is associated with cochlear presbycusis and its possible roles in delaying or preventing age-related hearing impairment. These studies not only provide novel insights into the molecular mechanisms underlying the regulation of spiral ganglion and SV cell survival with age, but could also lead to the development of novel therapeutic strategies for the prevention and treatment of ARHL, affecting many millions of people worldwide.

## RESULTS

### Aldosterone treatments prevent age-related hearing threshold elevations and improve hair cell responses

**Figure 1:**
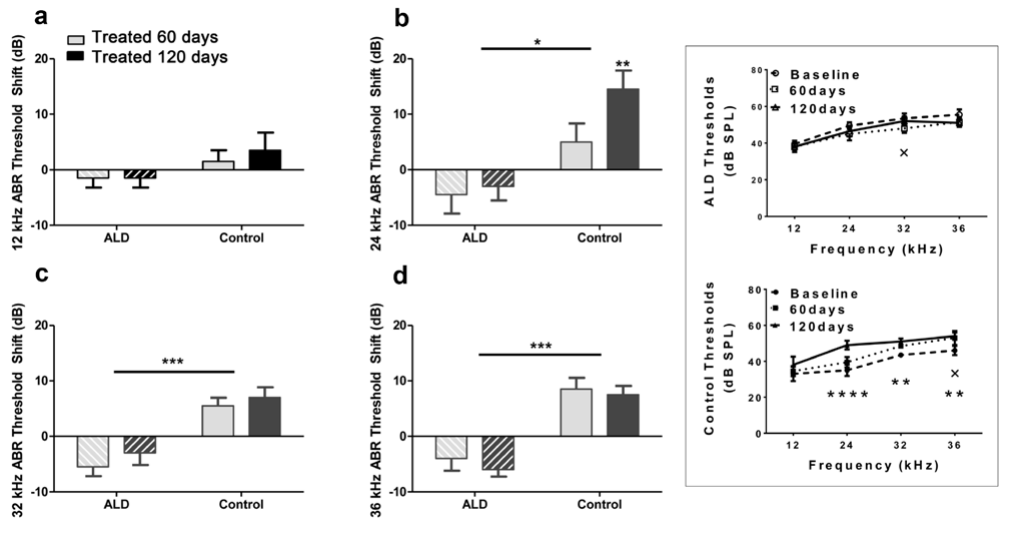
ABR threshold shifts (dB) for treated and untreated (control) subject groups following 60 and 120 days of treatment 12 kHz (**a**), 24 kHz (**b**), 32 kHz (**c**) and 36 kHz (**d**). Mean (±SEM) frequency-specific ABR threshold shifts in the treatment group (n = 5) on days 60 and 120 compared to the control group (n=5) values, showed improved hearing sensitivity for all four test frequencies, with the greatest benefit at the higher frequencies. “0” on the ordinate represents the baseline (pre-treatment) ABR thresholds. So, negative threshold shifts represent improvements in hearing with time in the ALD treatment group, while positive shifts indicate age-related ABR threshold elevations in the control group. (**e**) ABR audiogram data upon which 1a – 1d are based. Top: Very little change in auditory sensitivity occurs in the ALD treated mice. Bottom: The control mice show typical age-related hearing loss threshold elevations over the 4-month treatment period. Graphs show means (±SEM); solid line is the pre-treatment baseline ABR audiogram; the dotted line is for 60 days, and the dashed line is for 120 days of treatment. ANOVA: +p<0.05 for 60 days; *p<0.05 for 120 days; **p<0.01 for 120 days, ****p<0.0001 for 120 days.

**Figure 2:**
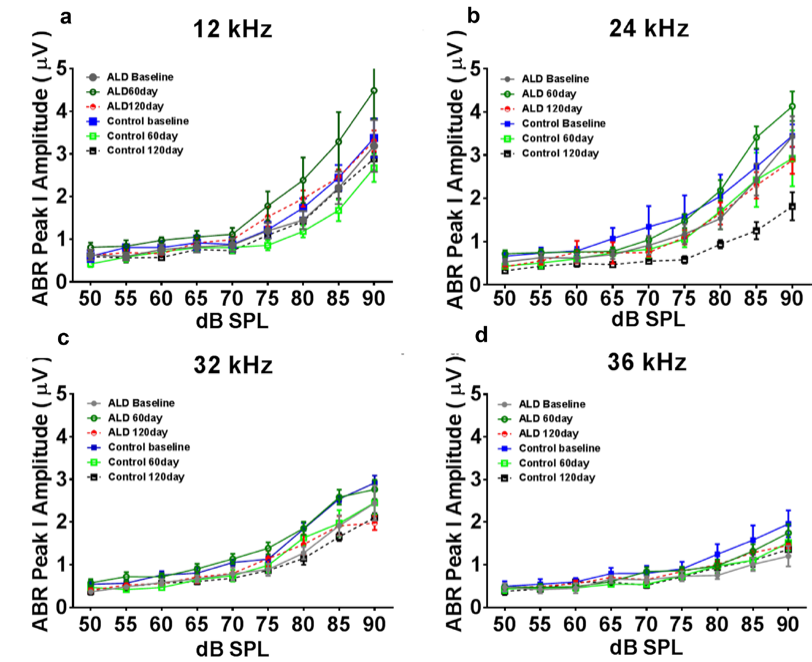
ABR Peak 1 amplitude changes (μV) as a function of sound intensity, for treated and untreated (control) subject groups, following 60 and 120 days of treatment 12 kHz (**a**), 24 kHz (**b**), 32 kHz (**c**) and 36 kHz (**d**). Note that the ALD treatment ABR levels tend to be above the control levels at 60 and 120 days of treatment, especially at the higher intensities, and for 12 and 24 kHz. Also, the control levels at the 120 day time point tend to be the lowest of all, due to the normal progression of age-related hearing loss. Quantification of these relative improvements in the ALD treatment groups for 80 dB SPL are given in the next Figure. Error bars are SEM.

**Figure 3:**
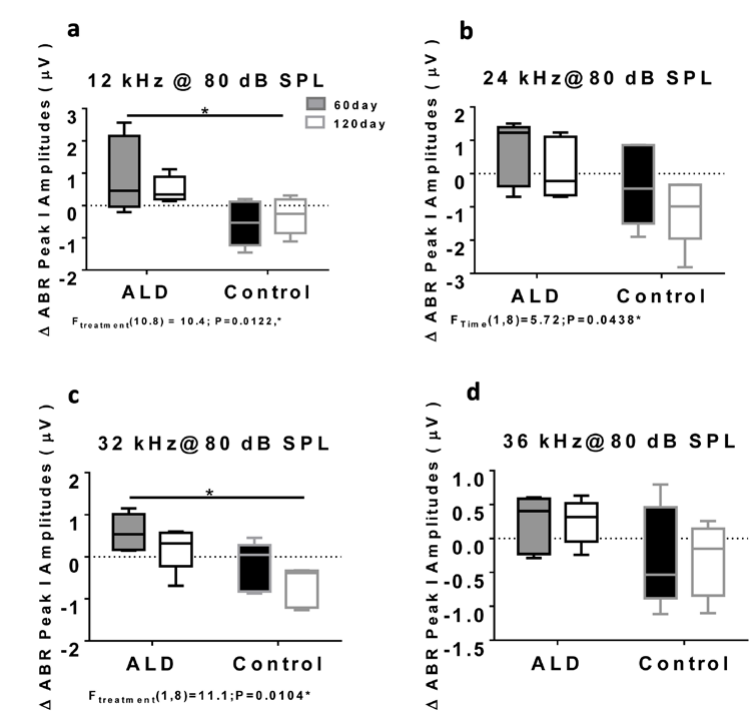
ABR Peak 1 amplitude changes (μV) at 80 dB SPL, for treated and untreated (control) subject groups, following 60 and 120 days of treatment 12 kHz (**a**), 24 kHz (**b**), 32 kHz (**c**) and 36 kHz (**d**). Box plots (median, 1st & 3rd quartiles, whiskers: minimum & maximum) of frequency-specific ABR P1 amplitude changes in the treatment group (n = 5) on days 60 and 120 compared to the control group (n=5) values, showed improved hearing, i.e., increased ABR amplitudes, for all four test frequencies, with the greater benefits at the higher intensities. “0” on the ordinate represents the baseline (pre-treatment) ABR amplitude levels. So, positive shifts represent increased excitatory drive with time in the ALD treatment group, while negative shifts indicate age-related ABR level decreases in the control group. ANOVA: *p<0.05; error bars are SEM.

First, standard hearing measurements were made to test the hypothesis that ALD may be beneficial for preventing or delaying age changes in the auditory system. The ALD pellet implantation was started at age 17 months, and lasted for 4 months. Long-term ALD treatment effects on peripheral hearing were assessed in mice by computing the ABR threshold shifts at 12, 24, 32 and 36 kHz and suprathreshold sound coding was measured with ABR peak amplitude intensity functions. Threshold improvements due to the ALD treatments were discovered at all frequencies with statistically significant improvements found when comparing the treated group to the non-treated control group (Figs. 1a-e). These data indicate that the maximum therapeutic effect is in the cochlear base, where ARHL typically exerts its maximal effects, with smaller improvements in the low frequency, apical region. In addition, ABR peak I amplitudes were found to be reduced at high intensities in the aging control group, but after long-term ALD treatment amplitudes were significantly increased, as displayed in Figures 2 and 3. Specifically, the increase in P1 amplitude from baseline controls is shown as a function of the intensity of the tone burst stimulus for 12, 24, 32 and 36 kHz, panels 2a-d, respectively. Improvements in threshold and suprathreshold benefits of the ALD were manifested at 60 days and became more prominent at 120 days of treatment, as shown for 80 dB in Figure 3 for all test frequencies, i.e., the ALD-treated ABR suprathreshold responses are higher than the controls. Also note that the treatments actually improved the ABR thresholds relative to the baseline thresholds that were measured just prior to the beginning of the treatments (baseline = 0 for the Figs. 1a-d ordinates; a negative shift indicates threshold improvements over the course of the treatments).

**Figure 4:**
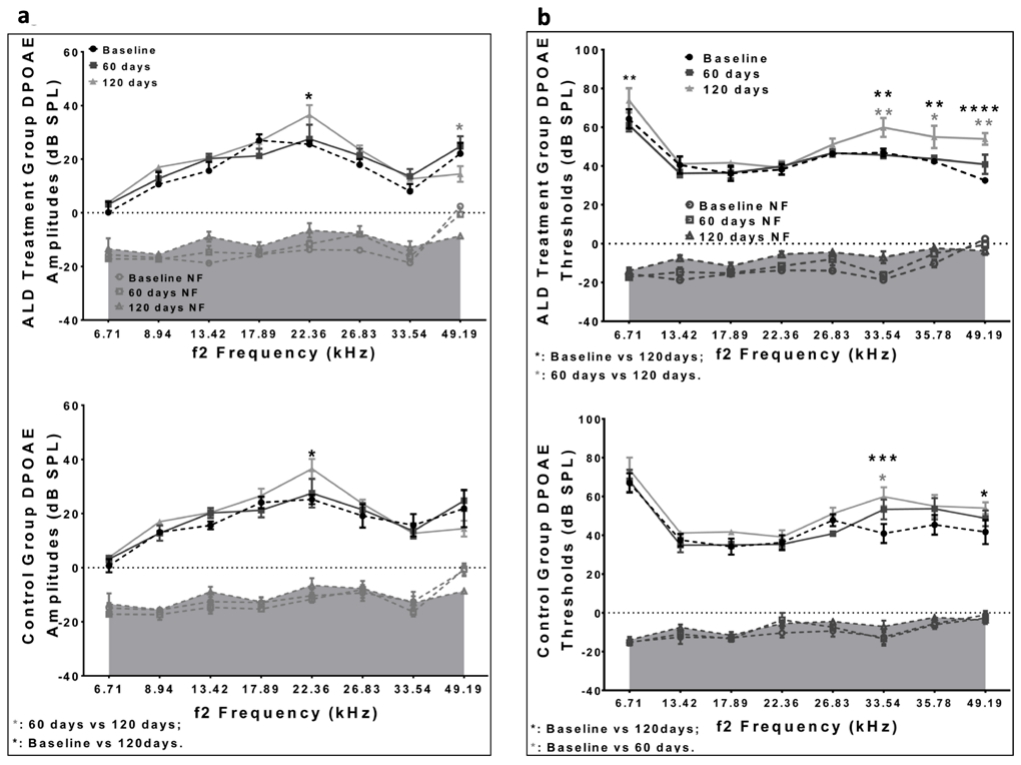
DPOAE amplitudes and thresholds for treated and untreated (control) subject groups, following 60 and 120 days of treatment (**a**) Amplitudes for the ALD (top) and Control (bottom) groups are similar to each other, and do not change systematically during the treatment period. (**b**) The DPOAE thresholds show improvement at the higher frequencies for the ALD treatment group at 60 days (top), but not for the Control group (bottom). Graphs show means (±SEM). ANOVA: *p<0.05; **p<0.01, ***p<0.001, ****p<0.0001.

Consistent with Schuknecht's classification scheme presented above ([8, 9]), ARHL in the CBA/CaJ mouse involves both hair cell and spiral ganglion neuron loss. Therefore, we made initial observations about the status of the outer hair cell system, by measuring distortion product otoacoustic emissions (DPOAEs) before and after ALD therapy. The results indicated an improvement in DPOAE thresholds in 21 month old mice with 60 day ALD treatments (Fig. 4). Based on these data, it appears that extended ALD treatments will significantly retard certain aspects of the aging process on hair cell loss; a finding worthy of more extensive investigation in subsequent studies.

### Serum aldosterone levels increased into the normal range in old mice with aldosterone therapy

**Figure 5:**
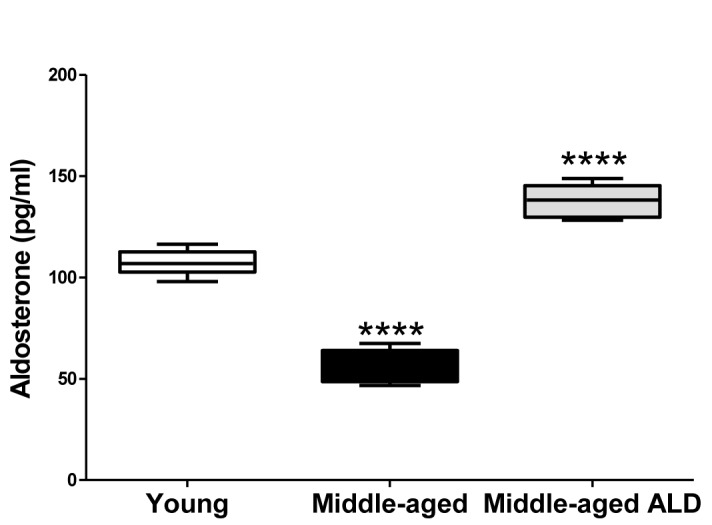
The serum aldosterone levels from young adult and middle age CBA/CaJ mice with or without the aldosterone treatment, 0.0016 mg/day continuous release, subcutaneous pellet through 120 days The systemic aldosterone treatment restored serum levels to near the normal range for young adults. Graphs show means (±SEM). ANOVA: ****p<0.0001.

Our preliminary findings in middle age CBA/CaJ mice provided an appropriate dosage for the longitudinal studies reported here [32]. In untreated aging mice, age-related declines of nearly 50% in serum ALD levels occur as compared to young adult mice (details presented in Fig. 5). However following treatments, serum ALD levels rose to near the normal range, very close to that of the young adult comparison group (Fig. 5). As we report in our companion article [33], ALD treatments did not elevate blood pressure; i.e., mean systolic (∼120 mm Hg) and diastolic (∼85 mm Hg) pressures remained stable relative to the baseline, and over the treatment periods of 60 and 120 days in both the control and treated groups. These results indicate that long-term ALD treatment does not induce a potential negative side effect, using the therapeutic dosing regimen of the present study.

### Aldosterone rescues age-related spiral ganglion cell loss

**Figure 6:**
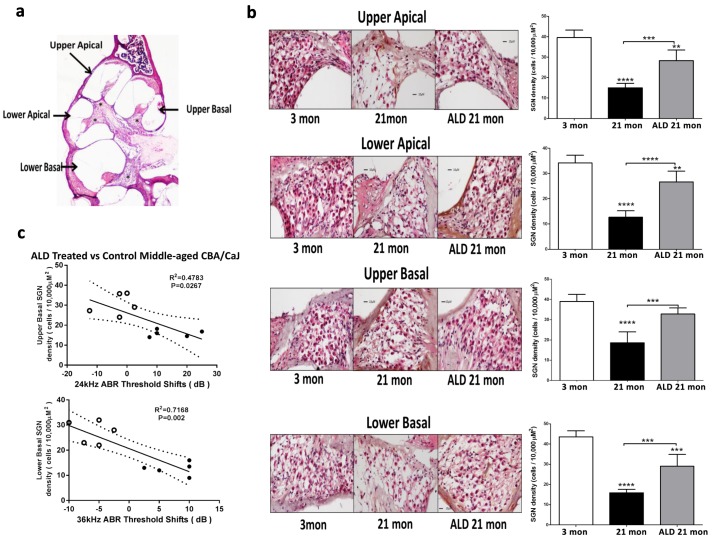
Hematoxylin/eosin (H&E) staining patterns in the cochlea reveal spiral ganglion neuron (SGN) loss with age, and rescue with aldosterone hormone treatments (**a**) Cross section of the complete CBA mouse cochlea showing all turns: Section-thickness is 5 μm, Magnification: 2.5 × 1.6. All cochlear turns are distinguishable as: upper apical, upper basal, lower apical and lower basal. The cell number measurements of SGNs included all turns: (**b**) SGN cell count densities were measured by light microscopy for the young adult (left panel) and middle-aged mice with (right panel) and without (middle panel) aldosterone treatments; Magnification: 20 × 1.6. Right panels show bar graphs representing the SGN cell density. Mean ±SEM for each subject group. *p<0.05, **p<0.01, ***p<0.001, ****p<0.0001. (**c**) Statistically significant correlations between SGN density and 24 and 36 kHz ABR threshold shifts indicate that a reduction in the number of SGNs are associated with poorer hearing (higher ABR thresholds). Open points are for ALD treated animals, and filled points are for the controls who are undergoing normal age-related hearing loss. According to the physiological place-frequency map of the mouse cochlea (see text), 24 and 36 kHz are located at upper and lower basal turns of the cochlea, which correspond to the cochlear locations with the most cell density loss with age in CBA/CaJ mice.

Interactions between improvements in hearing due to the ALD treatments and spiral ganglion neuron density were explored. Specifically, the spiral ganglion cell numbers in middle-age treated and control mice were compared to that of the untreated young adults. To accomplish this, cochlear cross sections were subjected to H&E staining and spiral ganglion cell counts were made for each cochlear turn (Fig. 6a). Mean cell counts/10,000 μm^2^ significantly decreased in the untreated middle-age mice (Figs. 6b), compared to the young adult mice. However, following long-term ALD treatment there was a noteworthy increase in the number of surviving spiral ganglion neurons, relative to age-matched controls, indicating a therapeutic effect at the level of the modiolus.

### Functional hearing preservation with aldosterone correlates with spiral ganglion cell rescue

In order to test for co-localization of spiral ganglion neuron rescue with frequency-specific hearing improvements following ALD treatments, the murine cochlear place-frequency map derived by Muller was utilized [34]. Müller and colleagues determined the cochlear location of physiologically characterized auditory nerve fibers along the cochlear partition and then mapped the location with best frequency [34, 35]. For example, a best frequency of 19.6 kHz was localized to a position 50.2% from the cochlear base, while 40.5 kHz is 18% from the basal end of the cochlea. In the present study, histological confirmation of the location of spiral ganglion neurons was correlated to ABR thresholds data. Specifically, relations between spiral ganglion neuron loss and ABR threshold shifts at 24 and 36 kHz were examined, and the results revealed a positive correlation between spiral ganglion neuron (SGN) density and ABR threshold shifts following 120 days of treatment with ALD: for 24 kHz vs upper basal SGN, r^2^ = 0.48, p = 0.027 (Fig. 6c, top); and for 36 kHz vs lower basal SGN, r^2^ = 0.72, p=0.002 (Fig. 6c, bottom). The rescue of SGNs provides anatomical confirmation that ALD has a protective effect on SGN survival with age and provides a basis for the maintenance of high frequency hearing ability.

### Mineralocorticoid receptors are expressed in the cochlea

**Figure 7:**
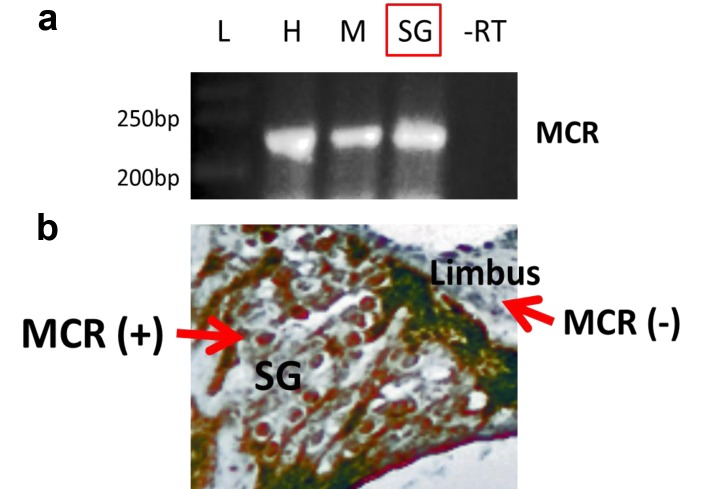
Mineralocorticoid receptor (MCR) expression and distribution in the cochlea of the young adult (3 mon) CBA/CaJ mouse (**a**) Multiplex RT-PCR was performed as described in the text. This experiment includes the absence of reverse transcription (control lane -RT). The mRNA expression of MCRs in tissue from heart (H), muscle (M) and spiral ganglion neurons (SG), where the product of RT-PCR is about 245 bp. -RT- reverse transcription without primers (as the negative control). L-Ladder: DNA molecular weight markers. (**b**) The protein expression of MCRs in SG was detected using immunohistochemistry staining (brown color, MCR+). A portion of the limbus is shown as a cochlear region not displaying significant MCR staining (MCR-).

Aldosterone is known to exert its physiological effects through the activation of mineralocorticoid receptors (MCRs). In general, MCRs are involved in the maintenance of cellular and structural integrity of neurons required for normal cognition, behavior and endocrine control [36]. Furthermore, MCRs are important for neuronal survival in the rat hippocampus, and increased survival of rat primary cortical neurons in response to mild stressors [28]. MCRs also improve survival of hippocampus neurons after transient global ischemia, and play regulatory roles in many other tissues [37, 38]. We hypothesized that like central nervous system neurons, SGNs would also be under the influence of the aldosterone-MCR axis. To test this, the expression patterns of MCRs in SGN cell bodies were examined. As a positive control RNA from mouse heart and liver were utilized, as MCRs are known to be abundant in cardiac and liver tissue [39-41]. A sample from the mouse heart without reverse transcription was used as negative control condition. Moderate MCR gene (Fig. 7, top) and protein (Fig. 7, bottom) expression was observed in the SGNs of young adults, and in stria vascularis cell bodies (not shown). More intense staining was observed in other cochlear structures such as the tectorial membrane.

### Aldosterone reverses the down-regulation of MCRs with age by blocking posttranslational modifications

**Figure 8:**
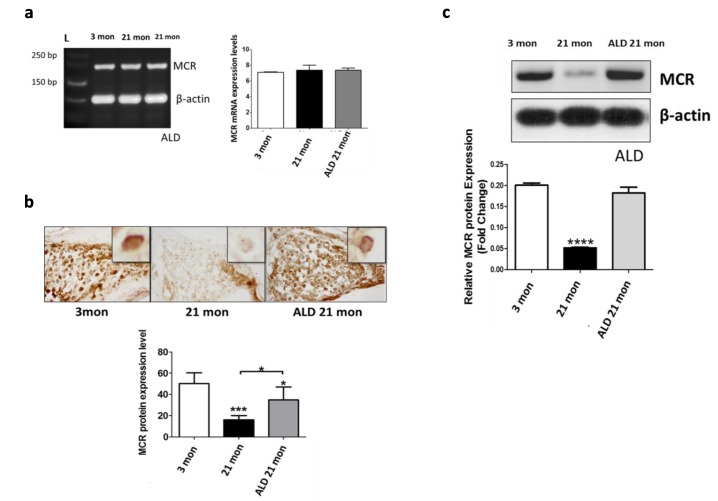
MCR presence in spiral ganglion neurons from young adult (3 mon), and aged (21 mon) control mice and those which were treated aldosterone (ALD) from 17 months up to 21 month (**a**) mRNA gene expression of MCRs in young adult, aged mice and aged mice with ALD treatment were all of similar magnitudes. 3 mon: Modiolar samples from young adult mice, 21 mon: Modiolar samples from aged mice. L-Ladder: DNA molecular weight. (**b**) The MCR protein expression level was determined by densitometry analysis (MetaMorph Image Analysis System) of immunocytochemistry sections; upper panels show representative sections for the MCR antibody staining. The insets in the upper panels represent a typical MCR-DAB stained spiral ganglion neuron. The lower panel presents a bar graph summarizing the relative densities from densitometry measurements: Mean ± SEM for each group. (**c**) MCR protein expression in spiral ganglion neurons shown by western blots of modiolar tissue samples. The expression level is reported relative to the expression of beta-actin as the loading control. For both measures of protein expression (b and c), the ALD treatments helped rescue the age-related declines in MCR protein expression. Statistical significance: *p< 0.05, **p<0.01, ***p<0.005, ****p<0.001.

MCR-mediated effects are largely independent of agonist ligands in transport epithelia such as the renal tubules, however this is not the case in neuronal systems, such as the brain, including the hippocampus, where modulation of the presence of MCR is ligand dependent [29, 30, 31]. As shown in Figure 8a, mRNA expression of MCRs in spiral ganglion neurons was detected, but there was no difference in the gene expression levels among all the subject groups. In contrast, both proteomics methodologies employed (immunohistochemistry, western blot), revealed that the MCR protein expression is down-regulated in spiral ganglion neurons of middle aged mice compared to young adults (Figs. 8b, 8c). However, following 4 months of ALD treatment a 2-fold up-regulation of MCR expression occurred. These results indicate that age-related down-regulation of MCRs in spiral ganglion cells involves a post-transcriptional modification that can be prevented by ALD hormone therapy initiated in middle age.

### Molecular mechanisms for age-linked spiral ganglion neuron death

**Figure 9:**
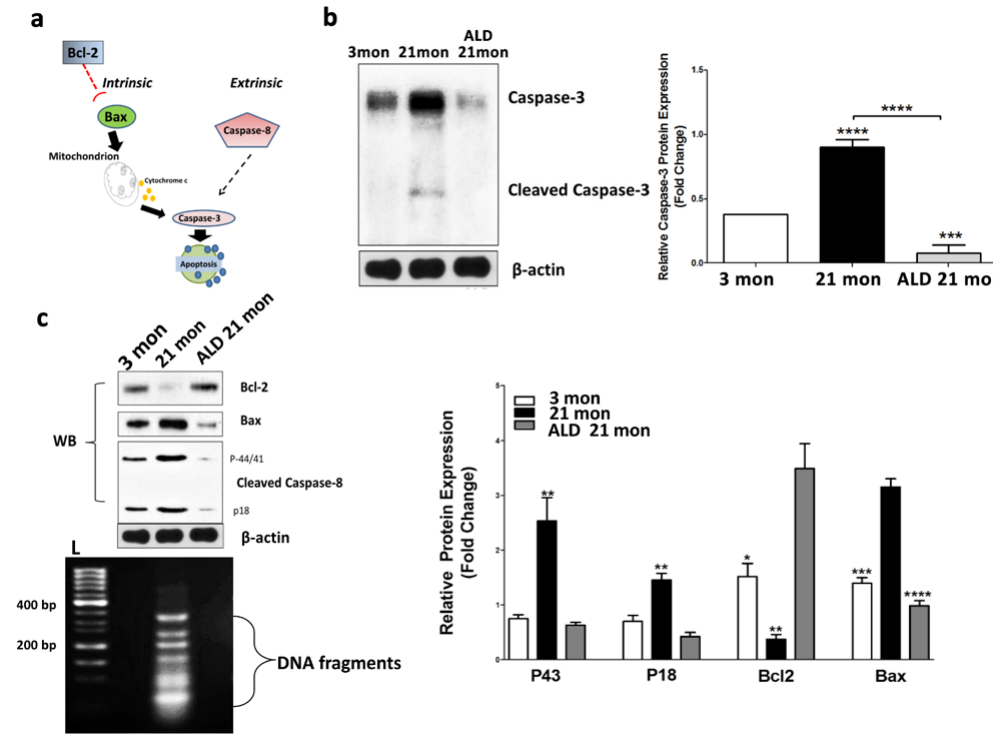
Apoptotic biomarker expression levels in modiolar tissue samples from young adult (2-3 mon), middle-aged (20-21 mon) ALD-treated and untreated CBA/CaJ mice Panel (**a**) shows the key molecules (Bcl-2, Bax, caspase 3 & 8) for the apoptotic pathways being investigated. (**b**) Cleaved caspase-3 protein expression is elevated in middle age controls, but reduced in ALD-treated mice. Since caspase-3 is an executor of apoptosis and a common factor of the intrinsic and extrinsic pathways, its upregulation can induce apoptosis. Note: the antibody utilized here also recognizes the cleaved caspase-3 to a lesser degree. Statistical differences are relative to the young adult levels. (**c**) To further assess age-related changes in the apoptotic pathways, the extrinsic factor, caspase-8, and intrinsic factors, Bcl-2 and Bax, were examined using western blots (WB). All three biomarkers were activated as shown: caspase-8 cleavages including p43 and p18 were enhanced in the middle age mice compared to the young adults, but reduced in ALD-treated mice. Bax was increased, but Bcl-2 was decreased with aging in SGNs, and ALD treatment inhibited the age-related changes of both molecules. Lastly, the DNA ladder detected in aging SGNs, but ALD treatment prevented the genomic DNA to be digested by endonucleases, L-Ladder: DNA molecular weight. *p<0.05, **p<0.01, ***p<0.001, ****p<0.0001.

One known mechanism by which MCRs can influence neuron survival in the brain is by inhibiting apoptosis [42]. In addition, we have previously identified age-linked upregulation of apoptotic pathways in the CBA/CaJ cochlea [43]. The signaling cascades inducing apoptosis in mammalian cells can be divided into two pathways: either mitochondrial (intrinsic) or death receptor (extrinsic) [44-46]. However, the extent to which apoptosis is a key mechanism for age-related spiral ganglion cell loss is not known. For instance, a previous investigation found no effects of Bax−/− or Bcl-2 over-expression on age-related loss of spiral ganglion neurons [47]. Based upon this study, and our previous work on cochlear apoptosis in CBA/CaJ mice [43], we further investigated the role that apoptotic pathways play in age-related spiral ganglion neuron loss. Pathways involved that may be independent of Bax/Bcl-2 pathways [46], are presented in Figure 9a. The intrinsic and extrinsic pathways, both at the end point of the execution phase, are often considered key components of the final apoptotic pathway. It is the activation of the execution caspases that begins this terminal phase of apoptosis. Execution caspases activate cytoplasmic endonucleases, which degrade nuclear material, and involve proteases that break down nuclear and cytoskeletal proteins. Caspase-3 is often considered to be the most important of the executioner caspases and is activated by any of the initiator caspases [48, 46]. We assayed the expression of Caspase-3 in spiral ganglion neurons to determine its involvement in ARHL. As shown in Figure 9b, Caspase-3 expression increased in middle-age spiral ganglion neurons compared to young adults, and the induction of cleaved caspase-3, which is the activated form of caspase-3, was also observed (Fig. 9b). Relevant to the activation of either intrinsic or extrinsic pathways, the apoptotic intrinsic inducer Bax was found to be upregulated, and the survivor factor Bcl-2 (likely involving inhibition of mitochondrial cytochrome c release) was down-regulated with aging in spiral ganglion neurons (Fig. 9c). These data suggest that the apoptotic intrinsic pathway is activated in cochlear aging. In addition, extrinsic factor Caspase-8 (a hallmark of apoptotic extrinsic pathways such as caspase-1, 3, 6, 7 chain reactions), activated two caspase-8 fragments, p18 and p43, in spiral ganglion neurons of middle age mice compared to the young adult group. In addition, following the 4 months of ALD treatment Caspase-3 expression levels were significantly reduced, and simultaneously the induction of cleaved caspase-3 disappeared (Fig. 9b, Western blot). Also, the declines of Bcl-2 with age were reversed, and the levels of activated Bax and caspase-8 were decreased with the ALD treatment (Fig. 9c). So together, these data indicate that the extrinsic and intrinsic apoptotic pathways are upregulated with age in spiral ganglion neurons, and ALD can be a double-edged intervention to block age-related apoptosis. These studies support the hypothesis that age-related cochlear neuron loss involves both extrinsic and intrinsic apoptotic pathways.

Additionally, apoptotic DNA fragmentation is a key feature of apoptosis, characterized by the activation of endogenous endonucleases with subsequent cleavage of chromatin DNA into internucleosomal fragments of roughly 180 base pairs (bp) and multiples thereof. DNA fragmentation can be detected via DNA laddering and the TUNEL assay. However, nucleosomal ladders have only been clearly demonstrated when vast numbers of cells die synchronously, so asynchronous cell death with subtle DNA fragmentation cannot be routinely detected by DNA fragmentation assays [49]. Therefore, we used a highly sensitive method, ligation-mediated polymerase chain reactions (LMPCR), to amplify the nucleosomal ladder of SGNs from young and middle-aged mice [49, 50, 51, 52-55]. Typically, DNA fragments have 5′-phosphorylated blunt ends, which can be ligated to dephosphorylated adaptors composed of a 12-mer and a 27-mer. When the mixture of ligated DNA fragments is heated, the 12-mer is released. Next, the 5′ protruding ends are filled in by a thermostable DNA polymerase. The 27-mer then serves as a primer in a PCR in which the fragments with adaptors on both ends are exponentially amplified. The resulting nucleosomal ladder can be easily visualized on an agarose/EtBr gel. The observed increase in apoptosis in aged spiral ganglion neurons was demonstrated by the elevated levels of cleaved genomic DNA by formation of a DNA ladder (Fig 9c), but ALD treatment for 4 months inhibits the aging induced DNA fragmentation ladder in spiral ganglion neurons

## DISCUSSION

Consistent with the success of the therapeutic intervention administered to middle age animals to slow down the progression of ARHL in the present report, there is growing evidence that key aspects of ARHL emerge in middle age. For example, the auditory efferent feedback system, known as the medial olivocochlear bundle, arises from the superior olivary complex of the brainstem and provides efferent control to the cochlear outer hair cells, providing a gain control mechanism for regulating hearing sensitivity in the presence of suprathreshold stimuli and background noise conditions [56, 57]. We previously discovered in middle-aged humans [58] and middle-aged mice [59-61], that this feedback loop starts to show significant functional declines. In addition, prevailing evidence indicates that auditory spectral and temporal processing deficits start to manifest themselves in middle age humans [62-64], and in animal models starting in middle age [65, 66]. Additional investigations, across the lifespan of CBA/CaJ mice, including young adult (up to 10 mon), middle age (10-20 mon) and old age (20- 30 mon) [67, 68], provide additional instances of relevant auditory functional deficits that start in middle-aged mice, including elevations in ABR thresholds, especially at the higher frequencies.

**Figure 10:**
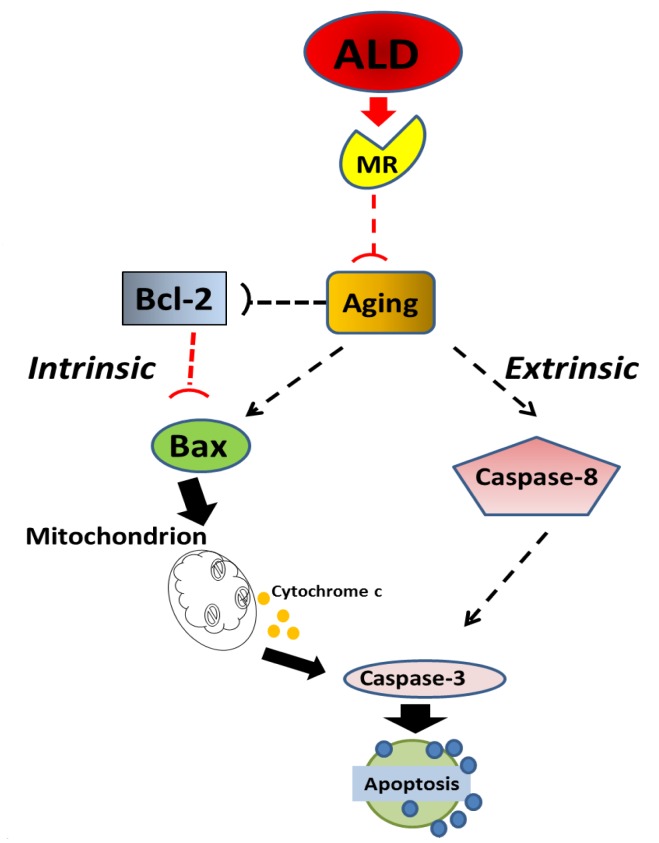
Schematic of the potential molecular pathways which illustrate the main findings of the present investigation, suggesting that ALD interferes with apoptotic pathways for aging in spiral ganglion neurons Aging processes triggers Bcl-2 inhibition and Bax activation, which are key factors in intrinsic pathways, and promotes the caspase-dependent extrinsic pathway. Aldo-sterone, through the activation of cochlear mineralocorticoid receptors, blocks apoptotic processing induced by aging, i.e., Bcl-2 is increased and Bax is decreased to inhibit the intrinsic apoptosis induced by aging, Caspase-dependent pathways are also inhibited.

There is also evidence to support possible roles of apoptosis in the aging cochlea, as has come from reports of the association between aging changes in apoptotic gene expression [43] and alterations in the expression of apoptosis-related proteins [69, 70]. For example, investigation of a role for apoptosis in hair cell and SGN loss with age has occurred using the terminal deoxynucleotidyl transferase-mediated dUTP-biotin nick end-labeling (TUNEL) method, and the presence of DNA fragmentation in hair cells and SGNs during aging has been found [71-73]. However, it is known that TUNEL is not quite specific for apoptotic cells even though it is a very popular method to detect apoptosis, as nuclear fragments from necrotic or autolytic cells may also be TUNEL-positive. The occurrence of false positive staining has been noticed for several conditions such as inappropriate fixation and unmasking after formalin fixation and paraffin embedding through protease addition [74-76]. More recently, the internucleosomal DNA cleavage pattern has been demonstrated as a specific feature of apoptosis, and has become a recognized marker of programmed cell death. However, in conditions in which only a small percentage of cells are apoptotic, or in which apoptosis is occurring asynchronously, genomic DNA ladders may appear as a smear and not be definitive. So, the ligation-mediated PCR is an option to amplify the nucleosomal ladder, increasing the visualization. Figure 10 summarizes the molecular cascade in which aging promotes the onset of apoptosis in SGNs, including both intrinsic (Bcl-2, Bax) and extrinsic (Caspases-dependent) pathways. However, the age-related involvement of apoptotic pathways in SGN cell loss in the aged cochlea is still not completely understood. For example, loss of hair cells and SGNs is still found in young adult mice lacking caspase-3, a key downstream caspase in the apoptotic cascade. So, it is likely that activated caspase-3 may be sufficient but not essential for the death of hair cells and SGNs [77]. For example, using both Bax knockout (Bax−/−) and Bcl-2 over-expressing mice, previous investigators failed to find any significant difference in age-related loss of hair cells and SGNs between controls and transgenic mice [47]. Taken together, the results of the present report and previous studies suggest that age-related hearing loss does not occur through an apoptotic pathway involving key members of the Bcl-2 family. However, it is likely that aging initiates apoptosis processing in an inner loop network, so inhibiting several key molecules, but not all apoptotic pathways. According to the findings of the present report, ALD interferes with the ageing process involving several molecules, such as Bcl-2 induction, and Bax and caspase pathway inhibition. Lastly, since mitochondrial activity is involved in these apoptotic pathways (Fig. 10), changes in oxidative stress and their therapeutic regulation may play a role here in modulating age-related hearing conditions [78]. In sum, these findings suggest that developing a biomedical intervention to be administered in middle age, would be an optimal time point to prevent or decrease salient features of ARHL.

In closing, the auditory system provides a unique window into the neurobiological changes that occur in the aging nervous system. Hearing and auditory perceptual capabilities can be tested quickly and quantitatively, so that correlations between sensory perception and anatomical and molecular biomarkers can be compared quantitatively, as was accomplished in the current investigation. For example, in the present study we showed that ALD treatments over a 4-month period could help preserve hearing in aging mice, and identified several key biomarkers, including mineralocorticoid receptors on SGNs, which are related to both apoptotic intrinsic and extrinsic pathways. So the present investigation is an essential pre-clinical step forward. Verification of these findings in another mammalian species, and exploration of the possible therapeutic effects on additional biological mechanisms of ARHL, to justify trials in humans will be additional necessary steps to move towards FDA clinical trials. Translationally, it may be that ALD combined with other critical compounds could be given systemically (orally, time-release skin patch), if there are no side effects, or have beneficial systemic effects for an aging mammal. Alternatively, if side effects are detrimental (which was not the case in the current study, e.g., stable blood pressure throughout the treatment period), a medicine to improve hearing in the elderly could be given locally to the inner ear via microsystem delivery techniques that we are currently developing [79-82], or via transtympanic injections into the middle ear near the round window, perhaps using nanoparticles as drug carrying vehicles.

## CONCLUSION

The findings of the current report indicate that ALD, a naturally occurring hormone that declines with age, when prescribed in middle age, can prevent or slow down some of the key aspects of presbycusis in aging mice. Our new results point towards possible cellular and molecular mechanisms for this therapeutic effect: Upregulation of mineralocorticoid receptors via reduction of post-translational protein modifications, preservation of hair cells and spiral ganglion cells, and partial blocking of apoptotic and cell death pathways. These animal model findings pave the way for translational medicine development of an FDA approved drug to treat presbycusis, which affects hundreds of millions of people worldwide. It is likely that an approved drug to treat ARHL will be a cocktail of two or more therapeutic agents, and based upon the discoveries in the current report, ALD will be one of those key compounds.

## MATERIALS AND METHODS

### Subjects

Fifteen CBA/CaJ young adult (2-3 mon) and middle-aged mice (16-17 mon) served as subjects. Original breeding pairs were purchased from Jackson Labs (Bar Harbor, ME), and used to provide an in-house source of subjects that were housed and maintained in a standard husbandry facility of the University of South Florida Vivarium. All procedures were compliant with the University of South Florida IACUC protocols and all federal animal welfare regulations, including the NIH Guide for the Care and Use of Laboratory Animals.

### Hearing measurements

The ABR and DPOAE testing procedures were similar to our previous reports [59, 61, 68].

#### Auditory brainstem response (ABR) procedures

Middle-aged mice were divided into two groups: treatment (N=5) and control (N=5), and had clearly visualized, healthy tympanic membranes. Prior to recording the auditory brainstem response (ABR) mice were anesthetized with a mixture of ketamine/xylazine (120 and 10 mg/kg body weight, respectively, IP injection). Briefly, ABR sessions were carried out in a soundproof acoustic chamber (IAC lined with Sonex), with body temperature maintained at 37°C with a heating pad. Prior to recording, the stimulus probe was placed near the tympanic membrane with the aid of an operating microscope. Needle electrodes were inserted at the vertex (non-inverted) and in the muscle posterior to the left pinna (inverted), with a ground inserted under the contralateral pinna. ABR waveforms were evoked with 5 msec tone pips (0.5- msec rise-fall times) with a cos^2^ onset envelope, delivered at 21/sec though electrostatic speakers (TDT EC1) connected by 4 cm tubes to the opening of the external ear canal. The response was amplified (10,000 X), filtered (100 Hz–3 kHz), and averaged using the BioSig (TDT, Gainesville, FL) data acquisition system. A total of 200 responses were averaged (with stimulus polarity alternated), using an ‘artifact reject’ algorithm, whereby response waveforms were discarded when peak-to-peak amplitude exceeded 7 μV, to prevent contamination by muscle activity. Intensity was varied in 5 dB steps starting at 80 dB and decreasing to at least 20 dB below threshold for a specific test frequency. Each intensity was replicated and threshold was defined as the lowest intensity at which a response was replicated, as determined by two experimenters blind to the experimental condition. The recording sessions occurred every 2 months after the initial evaluation (baseline) for a period of 4 months.

#### Distortion product otoacoustic emissions (DPOAEs)

Ipsilateral acoustic stimulation and simultaneous measurement of DPOAEs were accomplished with the Tucker Davis Tech. (TDT) BioSig III system. Stimuli were digitally synthesized at 200 kHz using SigGen software applications with the ratio of frequency 2 (F2) to frequency 1 (F1) constant at 1.25; L1 was equal to 65 dB sound pressure level (SPL) and L2 was equal to 50 dB SPL as calibrated in a 0.1-mL coupler simulating the mouse ear canal. After synthesis, F1, F2, were each passed through an RP2.1 D/A converter to PA5 programmable attenuators. Following attenuation, the signals went to ED1 speaker drivers which fed into the EC1 electrostatic loudspeakers coupled to the ear canal through short, flexible tubes with rigid plastic tapering tips. For DPOAE measurements, resultant ear canal sound pressure was recorded with an ER10B+ low-noise microphone and probe (Etymotic) housed in the same coupler as the F1 and F2 speakers. The output of the ER10B+ amplifier was put into an MA3 microphone amplifier, whose output went to an RP2.1 A/D converter for sampling at 200 kHz. A fast Fourier transform (FFT) was performed with TDT BioSig software on the resultant waveform. The magnitude of F1, F2, the 2f1-f2 distortion product, and the noise floor of the frequency bins surrounding the 2f1-f2 components were measured from the FFT. The procedure was repeated for geometric mean frequencies ranging from 5.6 to 44.8 kHz (eight frequencies per octave) to adequately assess the neuroethologically functional range of mouse hearing.

Mice were anesthetized with a mixture of ketamine and xylazine (120 and 10 mg/kg body weight, respectively) by intraperitoneal injection before all experimental sessions. All recording sessions were completed in a soundproof acoustic chamber (lined with Sonex) with body temperature maintained with a heating pad. Before recording, the operating microscope (Zeiss) was used to place the stimulus probe and microphone in the test ear close to the tympanic membrane. The recording session duration was limited by depth of anesthesia, and lasted approximately 1 hour per animal.

### Aldosterone hormone treatment implantation

After baseline ABR recording at 16-17 months of age, while still fully anesthetized, mice were assigned randomly to the treatment or control groups; the treatment group received implantation injection of a custom d-aldosterone pellet: 0.0016 mg/day, 120-day release (Innovative Research of America, Sarasota, FL), and the control group received the saline placebo, both placements were subcutaneous, just behind the shoulder. Blood Pressure Measurements: As a measure of cardiovascular health, a group of young adult (N=5) CBA/CaJ mice, and the middle-aged mice described above were placed in a restraining tube to acclimate them to having their blood pressure (BP) measured, by placing them in a holder for 15 min for 3 consecutive days prior to the actual BP measurements (Kent Scientific CODA™ tail-cuff system). The animal was either placed in the holder by picking up the tail, or the animal entered freely. The rear hatch to the holder was carefully secured, and care taken to avoid pinching the tail or any other body parts while securing the rear hatch. The mouse was allowed to rest at least 5-minutes to acclimate to the holder.

### Serum collection and anatomical tissue preparation

After 4 mon of ALD treatments, the middle-aged and young adult mice were exsanguinated via cardiac vessel perfusion, with Euthasol® (0.22ml/kg). A thoracotomy was performed to gain access to the heart and the cardiac vessel was punctured by an 18G sterile hypodermic needle. Blood was allowed to free-flow from the puncture site and collected with a sterile Pasteur pipette, then transferred to an Eppendorf tube in a 37°C water bath for 30 min, centrifuged 2000 rpm for 25 min, and then the serum was taken off and stored at −80°C. The mouse was then decapitated, and the cochleae were quickly removed from the temporal bone and transferred into ice-cold DPBS (HyClone Lab Inc., Logan, UT) on ice to dissect the modiolus from one of the cochlea using a Zeiss stereomicroscope; and then it was placed into an Eppendorf tube in dry ice and transferred into −80°C in preparation for RT-PCR and Western blots.

### Immunohistochemistry

The other cochlea was placed in a glass vial with 10 ml fresh 4% paraformaldehyde (Thermo Scientific, Rockford, IL) in PBS (0.1 M, pH 7.6) overnight at 4°C. After fixation, the cochleae were washed 3 × 10 min in PBS and then decalcified in 20 ml 10% EDTA (ethylenediaminetetraacetic acid, Fisher Scientific, Pittsburgh, PA) in PBS at 4°C; the cochleae were checked daily until decalcification was complete (approximately 1 week). Next, they were washed 3 × 10 min in PBS, transferred into 10% and 20% sucrose (Acros, Geel, Belgium) in PBS for 2 hours each, then kept in 30% sucrose in PBS for overnight at 4°C until sinking. The cochleae were embedded into degassed OCT (Tissue-Tek, Torrance, CA) overnight at 4°C, orientated into the cryomold (Tissue-Tek) with OCT degassed for 1 hr, and then frozen at −80°C. Cryosectioning was performed at 5μm/section, and sections were mounted on glass slides.

#### Hematoxylin and Eosin (H&E) Staining

Cochlear slides were washed in dH_2_O for 1 min, and then stained for 5 min with the BBC Histo·Perfect™ H&E Stain kit (BBC Biochemical, Mt. Vernon, WA). Slides were rinsed with dH_2_O for 1 min, differentiated by BBC Acid wash for 1 min, and rinsed with dH_2_O for 1 min; then placed into BBC Blueing solution for 30 sec, and rinsed by dH_2_O for 1 min. Next, the slides went into 70% alcohol for 30 sec, stained with BBC Special Eosin II for 1 min, dehydrated with BBC S2*Histo 5 × 20 sec, and cleared in xylene and coverslipped using a permanent mounting media (Fisher Scientific, Pittsburgh, PA). Semi-automated cell counts were made using the MetaMorph imaging system with a Leica DMR microscope, using procedures similar to our previous investigations of the mouse aging auditory system [61, 68, 83].

### Receptor protein expression measured with immunohistochemistry

Mineralocorticoid Receptor (MCR): Cochlear section slides were washed in TBS twice for 5 min. They were then incubated for 10 min at room temperature in 3% H_2_O_2_, diluted in methanol, and washed with TBS for 2 × 5 min. Each section was treated with 100-400 μl blocking solution (0.3% triton- X 100 and 5% normal goat serum in TBS) for 1 hr at room temperature. Then removed from the blocking solution, with the addition of 100-400 μl anti-MCR (SC-25709, Santa Cruz Biotechnology, Dallas, TX) 1:25 diluted in blocking solution. Next, incubated overnight at 4°C. In the morning, the slides were washed in TBS 3 × 5 min, then Covered with 1-3 drops of signal stain boost IHC detection reagent (HRP, Rabbit, Cell Signaling, Danvers, MA) and incubated in a humidified chamber for 30 min at room temperature. Then, 3 × 5 min washes with TBS. Then 100-400 μl DAB was placed on each slide for intensification, then the slides were immersed in dH_2_O, and washed 2 × 5 min, followed by dehydration and coverslipping.

### ELISA for measuring serum aldosterone levels

50 μl serum samples were processed using an aldosterone ELISA kit (#1875, Alpha Diagnostic Int., San Antonio, TX) according to the manufacturers' protocol. An absorbance microplate reader (ELx800TM) and microplate data collection & analysis software (Gen5TM, BioTek, Winooski, VT) were used for data collection, with data reported as pg/ml. The expected young adult range for mice is 25-315 pg/ml.

### Gene expression - RT-PCR

Semi-quantitative RT-PCR analysis was performed as previously described [84, 85]. Briefly, total RNA was extracted using the RNAeasy Mini Kit (Qiagen, Valencia, CA). Tissue and cells were vortexed for 1 min to shear genomic DNA before loading onto the RNeasy mini columns, and then eluted in a minimum volume of 30 ml and a maximum volume of 2 × 50 ml RNAase-free water. RNA obtained with this procedure was essentially free of genomic DNA. 10 ng of RNA was reverse transcribed and cDNA was subjected to PCR amplification. A quantitative reverse-transcription polymerase chain reaction (qRT-PCR) was performed using the Enhanced Avian HS RT-PCR-100 Kit (HSRT20, Sigma, St. Louis, MO). The reverse transcript (RT) reaction took place at 45°C for 50 min. The competition between primer sets was excluded by adjusting the reaction condition. Then the RT products went to PCR amplification directly. A first cycle of 10 min at 95°C, 45 sec at 65°C and 1 min at 72°C was followed by 45 sec at 95°C, 45 sec at 65°C and 1 min at 72°C for 25 cycles (see below). The conditions were chosen so that none of the RNAs analyzed reached a plateau at the end of the amplification protocol, i.e. they were in the exponential phase of amplification. Each set of reactions always included a no-sample negative control. We also performed a negative control containing RNA instead of cDNA to rule out genomic DNA contamination. The PCR products were analyzed on agarose gels stained with Gel Red Nucleic Acid Stain (Biotium, Hayward, CA).

### Nucleosomal ladders detection

Nucleosomal ladders were revealed by a ligation-mediated polymerase chain reaction (LMPCR). Genomic DNA was isolated using GenElute TM Mammalian Genomic DNA Miniprep Kit (Sigma, St. Louis, MO) according to the procedure described by their standard protocol. A 0.5 μg/sample of the digested DNA template was used. The first step is the ligation of dephosphorylated adaptors (120-mer and 27-mer, provided in the kit) to the end of the DNA fragments. Mixed 5 μl 10x ligation buffer, genomic 0.5 μg, adaptor (10X) and H_2_O up to 50 μl, the reaction mixture was heated to 55°C for 10 min; and then the adaptor oligonucleotides were allowed to anneal by cooling to 10°C over 1 hour. Then 0.5 μl of T4 DNA ligase was added, and the reactions incubated at 16°C for 16 hours. DNA PCR was performed as follows: one denaturation cycle at 94°C for 5 min, followed by 35 cycles at 94°C for 1 min, 60°C for 1 min, and 72°C for 50 sec. In the last cycle, the products were kept at 72°C for an additional 5 min. One-fifth of each reaction was separated on a 1.5% agarose gel, stained with ethidium bromide and photographed. The LM-PCR was performed using the DNA Ladder Assay (MBI, San Francisco, CA). 50 μl LM-PCR reactions were prepared containing 1.0 μg/μl DNA from the diluted annealing/ligation reaction, and an additional 24 mer at 1.25 pmol/μl, 1× Taq DNA polymerase buffer (67 mM Tris-HCl pH 8.8, 16.6 mM [NH4]2SO4, 0.45% Triton X-100, 200μg/ml gelatin, 320 μM dATP, dTTP, dGTP, dCTP, 2 mM MgCl2 and 0.1 U/μl Taq polymerase as a Taq-antibody complex allowing a PCR hot-start. The Taq-antibody complex was prepared with ‘Jumpstart’ Taq antibody (Sigma-Aldrich, MO, USA) according to instructions, generating a complex at 0.83 (Taq) U/μl. LM-PCR reactions were heated in a DNA Engine to 94°C for 1 min to activate Taq polymerase and remove 12 mers, then ramped to 72°C for 10 min to re-anneal the target DNA and generate a complimentary sequence to the ligated 24 mers. PCR then proceeded over 30 cycles at 94°C for 1 min followed by annealing/extension at 72°C for 2 min, then completed with a single 72°C for 10 min extension. 11 μl of each reaction was electrophoresed on a 2% agarose/EtBr gel.

### Proteomics - Western blot analysis

Similar to our recent report ([84]), modiolus/spiral ganglion neuron tissue lysates were prepared in RIPA buffer (Pierce #89901, Thermo Scientific, Waltham, MA) with protease inhibitor cocktail (#78430, Thermo Scientific, Rockford, IL). Cells and tissue samples were homogenized in buffer, followed by centrifugation at 2000 X for 10 min at 4°C. Supernatants were loaded at 200 μg of protein/lane, after the protein concentrations were determined by the Bradford protein assay. Proteins were fractionated by SDS-PAGE gel electrophoresis and transferred to a PVDF blotting membrane. The blot was incubated with β-actin and primary antibodies for MCR, Bcl-2, Bax and Caspase-8 (Cell Signaling, Danvers, MA). The secondary antibody was horseradish peroxidase-conjugated goat anti-rabbit IgG (1:2000, Cell Signaling).

### Statistical analyses

GraphPad Prism (5.04, La Jolla, CA) was used for data analyses. One-way analysis of variance (ANOVA) was used for comparing each group such as young adults vs middle-aged with treatment and without treatment, for: BP, serum aldosterone levels, cell density, and genes and protein expression data. Two-way ANOVA with repeated measures was used to compare the ABR threshold shifts in the two middle-aged groups with treatment durations of 60 and 120 days. Bonferroni post-hoc tests corrected for multiple comparisons were used in cases where the ANOVA main effects were significant. Linear regression was used for measuring the correlations between ABR threshold shifts and cell density. The p<0.05 was used for the statistical significance criterion.

## References

[1] Frisina DR, Frisina RD (1997). Speech recognition in noise and presbycusis: relations to possible neural mechanisms. Hear Res.

[2] Walton JP, Frisina RD, O'Neill WE (1998). Age-related alteration in processing of temporal sound features in the auditory midbrain of the CBA mouse. J Neurosci.

[3] Walton JP, Simon H, Frisina RD (2002). Age-related alterations in the neural coding of envelope periodicities. J Neurophysiol.

[4] Frisina RD, Walton JP (2006). Age-related structural and functional changes in the cochlear nucleus. Hear Res.

[5] Engle JR, Tinling S, Recanzone GH (2013). Age-related hearing loss in rhesus monkeys is correlated with cochlear histopathologies. PLoS One.

[6] Omata Y, Tharasegaran S, Lim YM, Yamasaki Y, Ishigaki Y, Tatsuno T, Maruyama M, Tsuda L (2016). Expression of amyloid-β in mouse cochlear hair cells causes an early-onset auditory defect in high-frequency sound perception. Aging (Albany NY).

[7] Ding D, Jiang H, Chen GD, Longo-Guess C, Muthaiah VP, Tian C, Sheppard A, Salvi R, Johnson KR (2016). N-acetyl-cysteine prevents age-related hearing loss and the progressive loss of inner hair cells in γ-glutamyl transferase 1 deficient mice. Aging (Albany NY).

[8] Schuknecht HF, Gacek MR (1993). Cochlear pathology in presbycusis. Ann Otol Rhinol Laryngol.

[9] Schuknecht HF (1994). Auditory and cytocochlear correlates of inner ear disorders. Otolaryngol Head Neck Surg.

[10] Keithley EM, Feldman ML (1979). Spiral ganglion cell counts in an age-graded series of rat cochleas. J Comp Neurol.

[11] Keithley EM, Ryan AF, Woolf NK (1989). Spiral ganglion cell density in young and old gerbils. Hear Res.

[12] Schmiedt RA, Lang H, Okamura HO, Schulte BA (2002). Effects of furosemide applied chronically to the round window: a model of metabolic presbyacusis. J Neurosci.

[13] Schmiedt RA (1996). Effects of aging on potassium homeostasis and the endocochlear potential in the gerbil cochlea. Hear Res.

[14] Schmiedt R The Physiology of Cochlear Presbycusis.

[15] Ding B, Frisina RD, Zhu X, Sakai Y, Sokolowski B, Walton JP (2014). Direct control of Na(+)-K(+)-2Cl(−)-cotransport protein (NKCC1) expression with aldosterone. Am J Physiol Cell Physiol.

[16] Gratton MA, Smyth BJ, Lam CF, Boettcher FA, Schmiedt RA (1997). Decline in the endocochlear potential corresponds to decreased Na,K-ATPase activity in the lateral wall of quiet-aged gerbils. Hear Res.

[17] Ohlemiller KK, Dahl AR, Gagnon PM (2010). Divergent aging characteristics in CBA/J and CBA/CaJ mouse cochleae. J Assoc Res Otolaryngol.

[18] Tadros SF, Frisina ST, Mapes F, Frisina DR, Frisina RD (2005). Higher serum aldosterone correlates with lower hearing thresholds: a possible protective hormone against presbycusis. Hear Res.

[19] Trune DR, Kempton JB, Kessi M (2000). Aldosterone (mineralocorticoid) equivalent to prednisolone (glucocorticoid) in reversing hearing loss in MRL/MpJ-Fas1pr autoimmune mice. Laryngoscope.

[20] Trune DR, Kempton JB, Gross ND (2006). Mineralocorticoid receptor mediates glucocorticoid treatment effects in the autoimmune mouse ear. Hear Res.

[21] Trune DR, Kempton JB (2001). Aldosterone and prednisolone control of cochlear function in MRL/MpJ-Fas(lpr) autoimmune mice. Hear Res.

[22] Magdich LV (1980). [Age and the effect of adrenocorticotropic hormone on aldosterone secretion in rats]. Biull Eksp Biol Med.

[23] Hegstad R, Brown RD, Jiang NS, Kao P, Weinshilboum RM, Strong C, Wisgerhof M (1983). Aging and aldosterone. Am J Med.

[24] Hallengren B, Elmståhl S, Galvard H, Jerntorp P, Manhem P, Pessah-Rasmussen H, Stavenow L (1992). 80-year-old men have elevated plasma concentrations of catecholamines but decreased plasma renin activity and aldosterone as compared to young men. Aging (Milano).

[25] Bauer JH (1993). Age-related changes in the renin-aldosterone system. Physiological effects and clinical implications. Drugs Aging.

[26] Belmin J, Lévy BI, Michel JB (1994). Changes in the renin-angiotensin-aldosterone axis in later life. Drugs Aging.

[27] Mulkerrin E, Epstein FH, Clark BA (1995). Aldosterone responses to hyperkalemia in healthy elderly humans. J Am Soc Nephrol.

[28] Kau MM, Chen JJ, Wang SW, Cho WL, Wang PS (1999). Age-related impairment of aldosterone secretion in zona glomerulosa cells of ovariectomized rats. J Investig Med.

[29] Joëls M, Karst H, DeRijk R, de Kloet ER (2008). The coming out of the brain mineralocorticoid receptor. Trends Neurosci.

[30] De Kloet ER, Versteeg DH, Kovacs GL (1983). Aldosterone blocks the response to corticosterone in the raphe-hippocampal serotonin system. Brain Res.

[31] Chao HM, Ma LY, McEwen BS, Sakai RR (1998). Regulation of glucocorticoid receptor and mineralocorticoid receptor messenger ribonucleic acids by selective agonists in the rat hippocampus. Endocrinology.

[32] Chao HM, Sakai RR, Ma LY, McEwen BS (1998). Adrenal steroid regulation of neurotrophic factor expression in the rat hippocampus. Endocrinology.

[33] Zhu X, Ding B, Walton JP, Frisina RD (2014). Aldosterone reduces spiral ganglion neuron loss in middle –aged CBA/CaJ mice. Assoc. Res. Otolaryngol. Abs.

[34] Halonen J, Hinton AS, Frisina RD, Ding B, Zhu X, Walton JP (2016). Long-term treatment with aldosterone slows the progression of age-related hearing loss. Hear Res.

[35] Müller M, von Hünerbein K, Hoidis S, Smolders JW (2005). A physiological place-frequency map of the cochlea in the CBA/J mouse. Hear Res.

[36] Meyer AC, Frank T, Khimich D, Hoch G, Riedel D, Chapochnikov NM, Yarin YM, Harke B, Hell SW, Egner A, Moser T (2009). Tuning of synapse number, structure and function in the cochlea. Nat Neurosci.

[37] Berger S, Wolfer DP, Selbach O, Alter H, Erdmann G, Reichardt HM, Chepkova AN, Welzl H, Haas HL, Lipp HP, Schütz G (2006). Loss of the limbic mineralocorticoid receptor impairs behavioral plasticity. Proc Natl Acad Sci USA.

[38] Funder JW (2010). Aldosterone and mineralocorticoid receptors in the cardiovascular system. Prog Cardiovasc Dis.

[39] Hawkins UA, Gomez-Sanchez EP, Gomez-Sanchez CM, Gomez-Sanchez CE (2012). The ubiquitous mineralocor-ticoid receptor: clinical implications. Curr Hypertens Rep.

[40] Latouche C, Sainte-Marie Y, Steenman M, Castro Chaves P, Naray-Fejes-Toth A, Fejes-Toth G, Farman N, Jaisser F (2010). Molecular signature of mineralocorticoid receptor signaling in cardiomyocytes: from cultured cells to mouse heart. Endocrinology.

[41] Lother A, Berger S, Gilsbach R, Rösner S, Ecke A, Barreto F, Bauersachs J, Schütz G, Hein L (2011). Ablation of mineralocorticoid receptors in myocytes but not in fibroblasts preserves cardiac function. Hypertension.

[42] Nagata K, Obata K, Xu J, Ichihara S, Noda A, Kimata H, Kato T, Izawa H, Murohara T, Yokota M (2006). Mineralocorticoid receptor antagonism attenuates cardiac hypertrophy and failure in low-aldosterone hypertensive rats. Hypertension.

[43] Crochemore C, Lu J, Wu Y, Liposits Z, Sousa N, Holsboer F, Almeida OF (2005). Direct targeting of hippocampal neurons for apoptosis by glucocorticoids is reversible by mineralocorticoid receptor activation. Mol Psychiatry.

[44] Tadros SF, D'Souza M, Zhu X, Frisina RD (2008). Apoptosis-related genes change their expression with age and hearing loss in the mouse cochlea. Apoptosis.

[45] Green DR (2000). Apoptotic pathways: paper wraps stone blunts scissors. Cell.

[46] Hengartner MO (2000). The biochemistry of apoptosis. Nature.

[47] Tait SW, Green DR (2010). Mitochondria and cell death: outer membrane permeabilization and beyond. Nat Rev Mol Cell Biol.

[48] Shen H, Matsui JI, Lei D, Han L, Ohlemiller KK, Bao J (2010). No dramatic age-related loss of hair cells and spiral ganglion neurons in Bcl-2 over-expression mice or Bax null mice. Mol Neurodegener.

[49] Elmore S (2007). Apoptosis: a review of programmed cell death. Toxicol Pathol.

[50] Staley K, Blaschke AJ, Chun J (1997). Apoptotic DNA fragmentation is detected by a semi-quantitative ligation-mediated PCR of blunt DNA ends. Cell Death Differ.

[51] Arends MJ, Morris RG, Wyllie AH (1990). Apoptosis. The role of the endonuclease. Am J Pathol.

[52] Barnes WM (1994). PCR amplification of up to 35-kb DNA with high fidelity and high yield from lambda bacteriophage templates. Proc Natl Acad Sci USA.

[53] Wyllie AH (1980). Glucocorticoid-induced thymocyte apoptosis is associated with endogenous endonuclease activation. Nature.

[54] Cheng S, Fockler C, Barnes WM, Higuchi R (1994). Effective amplification of long targets from cloned inserts and human genomic DNA. Proc Natl Acad Sci USA.

[55] Hooker DJ, Gorry PR, Ellett AM, Wesselingh SL, Cherry CL (2009). Measuring and monitoring apoptosis and drug toxicity in HIV patients by ligation-mediated polymerase chain reaction. J Cell Mol Med.

[56] Liu QY, Ribecco-Lutkiewicz M, Carson C, Testolin L, Bergeron D, Kohwi-Shigematsu T, Walker PR, Sikorska M (2003). Mapping the initial DNA breaks in apoptotic Jurkat cells using ligation-mediated PCR. Cell Death Differ.

[57] Guinan JJ (2006). Olivocochlear efferents: anatomy, physiology, function, and the measurement of efferent effects in humans. Ear Hear.

[58] Liberman MC, Guinan JJ (1998). Feedback control of the auditory periphery: anti-masking effects of middle ear muscles vs. olivocochlear efferents. J Commun Disord.

[59] Kim S, Frisina DR, Frisina RD (2002). Effects of age on contralateral suppression of distortion product otoacoustic emissions in human listeners with normal hearing. Audiol Neurootol.

[60] Frisina RD, Newman SR, Zhu X (2007). Auditory efferent activation in CBA mice exceeds that of C57s for varying levels of noise. J Acoust Soc Am.

[61] Jacobson M, Kim S, Romney J, Zhu X, Frisina RD (2003). Contralateral suppression of distortion-product otoacoustic emissions declines with age: a comparison of findings in CBA mice with human listeners. Laryngoscope.

[62] Zhu X, Vasilyeva ON, Kim S, Jacobson M, Romney J, Waterman MS, Tuttle D, Frisina RD (2007). Auditory efferent feedback system deficits precede age-related hearing loss: contralateral suppression of otoacoustic emissions in mice. J Comp Neurol.

[63] Fitzgibbons PJ, Gordon-Salant S (2010). Age-related differences in discrimination of temporal intervals in accented tone sequences. Hear Res.

[64] Hall JW, Buss E, Grose JH, Roush PA (2012). Effects of age and hearing impairment on the ability to benefit from temporal and spectral modulation. Ear Hear.

[65] Snell KB, Mapes FM, Hickman ED, Frisina DR (2002). Word recognition in competing babble and the effects of age, temporal processing, and absolute sensitivity. J Acoust Soc Am.

[66] Leong UC, Barsz K, Allen PD, Walton JP (2011). Neural correlates of age-related declines in frequency selectivity in the auditory midbrain. Neurobiol Aging.

[67] Williamson TT, Zhu X, Walton JP, Frisina RD (2015). Auditory brainstem gap responses start to decline in mice in middle age: a novel physiological biomarker for age-related hearing loss. Cell Tissue Res.

[68] Guimaraes P, Zhu X, Cannon T, Kim S, Frisina RD (2004). Sex differences in distortion product otoacoustic emissions as a function of age in CBA mice. Hear Res.

[69] Zettel ML, Zhu X, O'Neill WE, Frisina RD (2007). Age-related decline in Kv3.1b expression in the mouse auditory brainstem correlates with functional deficits in the medial olivocochlear efferent system. J Assoc Res Otolaryngol.

[70] Alam SA, Oshima T, Suzuki M, Kawase T, Takasaka T, Ikeda K (2001). The expression of apoptosis-related proteins in the aged cochlea of Mongolian gerbils. Laryngoscope.

[71] Nevado J, Sanz R, Casqueiro JC, Ayala A, García-Berrocal JR, Ramírez-Camacho R (2006). Ageing evokes an intrinsic pro-apoptotic signalling pathway in rat cochlea. Acta Otolaryngol.

[72] Jókay I, Soós G, Répássy G, Dezsõ B (1998). Apoptosis in the human inner ear. Detection by in situ end-labeling of fragmented DNA and correlation with other markers. Hear Res.

[73] Someya S, Yamasoba T, Weindruch R, Prolla TA, Tanokura M (2007). Caloric restriction suppresses apoptotic cell death in the mammalian cochlea and leads to prevention of presbycusis. Neurobiol Aging.

[74] Usami S, Takumi Y, Fujita S, Shinkawa H, Hosokawa M (1997). Cell death in the inner ear associated with aging is apoptosis?. Brain Res.

[75] Garrity MM, Burgart LJ, Riehle DL, Hill EM, Sebo TJ, Witzig T (2003). Identifying and quantifying apoptosis: navigating technical pitfalls. Mod Pathol.

[76] Lawrence MD, Blyth BJ, Ormsby RJ, Tilley WD, Sykes PJ (2013). False-positive TUNEL staining observed in SV40 based transgenic murine prostate cancer models. Transgenic Res.

[77] Negoescu A, Lorimier P, Labat-Moleur F, Drouet C, Robert C, Guillermet C, Brambilla C, Brambilla E (1996). In situ apoptotic cell labeling by the TUNEL method: improvement and evaluation on cell preparations. J Histochem Cytochem.

[78] Takahashi K, Kamiya K, Urase K, Suga M, Takizawa T, Mori H, Yoshikawa Y, Ichimura K, Kuida K, Momoi T (2001). Caspase-3-deficiency induces hyperplasia of supporting cells and degeneration of sensory cells resulting in the hearing loss. Brain Res.

[79] Kwon DN, Park WJ, Choi YJ, Gurunathan S, Kim JH (2015). Oxidative stress and ROS metabolism via down-regulation of sirtuin 3 expression in Cmah-null mice affect hearing loss. Aging (Albany NY).

[80] Borkholder DA, Zhu X, Frisina RD (2014). Round window membrane intracochlear drug delivery enhanced by induced advection. J Control Release.

[81] orkholder DA, Zhu X, Hyatt BT, Archilla AS, Livingston WJ, Frisina RD (2010). Murine intracochlear drug delivery: reducing concentration gradients within the cochlea. Hear Res.

[82] Johnson DG, Frisina RD, Borkholder DA (2011). In-plane biocompatible microfluidic interconnects for implantable microsystems. IEEE Trans Biomed Eng.

[83] Johnson DG, Waldron MJ, Frisina RD, Borkholder DA (2010). Implantable micropump technologies for murine intracochlear infusions. Conf Proc IEEE Eng Med Biol Soc.

[84] Tang X, Zhu X, Ding B, Walton JP, Frisina RD, Su J (2014). Age-related hearing loss: GABA, nicotinic acetylcholine and NMDA receptor expression changes in spiral ganglion neurons of the mouse. Neuroscience.

[85] Marone M, Mozzetti S, De Ritis D, Pierelli L, Scambia G (2001). Semiquantitative RT-PCR analysis to assess the expression levels of multiple transcripts from the same sample. Biol Proced Online.

